# Inflammatory Myofibroblastic Tumor of the Hard Palate and Gingivobuccal Sulcus Mimicking a Malignant Neoplasm: A Case Report

**DOI:** 10.7759/cureus.108526

**Published:** 2026-05-08

**Authors:** Vaishnavi N Setloor, Sahana Srinath, Aryan Wadehra, Akhila S, Lakshmi Bagoor

**Affiliations:** 1 Oral Pathology and Microbiology, Government Dental College and Research Institute, Bengaluru, IND

**Keywords:** diagnostic sampling dilemma, hard palate, inflammatory myofibroblastic tumor, oral pathology, spindle cell lesion

## Abstract

Inflammatory myofibroblastic tumor (IMT) is an uncommon mesenchymal neoplasm of intermediate biological potential with rare occurrence in the oral cavity. Its broad histopathologic spectrum, coupled with variable immunohistochemical expression, renders it a significant diagnostic challenge, particularly in elderly patients with oral risk habits where malignancy is the primary clinical concern. We report a case of anaplastic lymphoma kinase (*ALK*)-negative IMT of the myxoid pattern arising simultaneously in the right hard palate and gingivobuccal sulcus in a 75-year-old female with a long-standing history of betel quid chewing. The patient was referred from a tertiary cancer center with a strong clinical and radiologic suspicion of malignancy based on rapid growth, ulceration, and contrast-enhanced CT demonstrating an ill-defined heterogeneously enhancing lesion with alveolar ridge erosion. Two successive biopsies were non-diagnostic, revealing only granulation tissue and fibroblastic proliferation. Subsequent deeper sections and an extended immunohistochemical panel demonstrated diffuse positivity for smooth muscle actin in the characteristic myofibroblastic tram-track staining pattern, desmin, and vimentin, with negativity for *ALK* and CD34. Focal cytokeratin positivity was noted but was not interpreted as evidence of epithelial malignancy in the absence of diffuse epithelial marker expression. Complete surgical excision was performed with clear margins, and the patient remained recurrence-free at five-month follow-up. This case illustrates the compounding diagnostic pitfalls of oral IMT: dual-site involvement, two failed biopsies, *ALK* negativity in an elderly patient, and focal cytokeratin positivity risking misclassification as spindle cell carcinoma. Awareness of IMT histologic subtypes, meticulous tissue sampling, and systematic immunohistochemical profiling are essential to avoid misdiagnosis and unnecessary aggressive oncologic treatment.

## Introduction

Inflammatory myofibroblastic tumor (IMT) is an uncommon soft-tissue neoplasm of intermediate biological potential, characterized by a proliferation of myofibroblasts accompanied by a variably dense inflammatory infiltrate composed of plasma cells, lymphocytes, and eosinophils [1]. Although IMT most frequently arises in the lung, mesentery, and abdominopelvic regions, involvement of the head and neck is rare, with the oral cavity being an exceptionally uncommon site of occurrence. Head and neck IMTs account for approximately 14%-18% of all extrapulmonary cases, with oral cavity involvement reported in fewer than 5% of head and neck cases [2].

Histopathologically, IMT is classified into three patterns based on the landmark classification of Coffin et al.: (1) a myxoid, vascular, and inflammatory pattern resembling nodular fasciitis; (2) a compact spindle cell pattern with intermingled inflammatory cells resembling fibrous histiocytoma; and (3) a hypocellular fibrous pattern with dense plate-like collagen resembling a desmoid or scar [3]. These overlapping patterns may coexist within a single tumor and frequently overlap with reactive granulation tissue, nodular fasciitis, fibromatosis, and low-grade spindle cell neoplasms [3]. In the oral cavity, IMT may present as a rapidly enlarging mass with ulceration or associated bone involvement, leading to clinical and radiologic suspicion of malignancy [4,5]. These features, coupled with its variable morphology, pose a significant diagnostic challenge for both clinicians and pathologists [6].

Immunohistochemistry plays a pivotal role in establishing the diagnosis, with tumor cells typically expressing smooth muscle actin characteristically in a myofibroblastic tram-track pattern, with desmin and vimentin positivity [7]. Anaplastic lymphoma kinase (*ALK*) gene rearrangements are identified in approximately 50%-60% of IMTs overall, predominantly in younger patients, while *ALK*-negative IMTs are more commonly encountered in adults and further complicate the diagnostic process [7-9]. Other molecular alterations, including ROS1 rearrangements and platelet-derived growth factor receptor β overexpression, have been identified in a subset of *ALK*-negative IMTs, further supporting a neoplastic pathogenesis.

We report an unusual case of *ALK*-negative IMT involving the right hard palate and gingivobuccal sulcus simultaneously in a 75-year-old female with a long-standing betel quid chewing habit. This combination of patient demographics, dual-site oral involvement, and risk habit background led to referral to a tertiary cancer center with a strong clinical and radiologic suspicion of malignancy. The diagnostic course was further complicated by two successive non-diagnostic biopsies and focal cytokeratin positivity on immunohistochemistry, both recognized but underappreciated pitfalls in the workup of oral spindle cell lesions. This case is reported to highlight these compounding diagnostic challenges, to reinforce the critical role of adequate tissue sampling and an extended immunohistochemical panel, and to draw attention to *ALK*-negative oral IMT as an entity that must be considered even in elderly patients with oral risk habits and radiologic features indistinguishable from malignancy [5,6].

## Case presentation

A 75-year-old female was referred from a tertiary cancer center for review of histopathological slides of a rapidly enlarging growth involving the right hard palate and maxillary vestibule. The lesion initially appeared as a small swelling in the right upper gingival region approximately one month before presentation and progressively increased in size. She also gave a history of a fall in a moving bus around the same time. Trauma has been proposed as a possible triggering factor for IMT in some cases, though a definitive causal relationship could not be established here.

The patient had a long-standing habit of betel quid chewing with tobacco and slaked lime for nearly 30 years, practiced five to six times daily, with habitual placement in the right buccal vestibule for approximately one hour each time. She had recently discontinued the habit. Associated symptoms included right-sided nasal obstruction and difficulty in mastication and swallowing. There was no history of systemic illness, prior malignancy, or medication use.

Clinical examination

Extraoral examination revealed bilateral palpable submandibular lymph nodes measuring approximately 1 × 1 cm. The right node was firm and mobile, while the left was soft and mobile; both were non-tender.

Intraorally, a well-defined, lobulated mass was noted on the right hard palate with extension into the right maxillary gingivobuccal sulcus. The palatal component measured approximately 6 × 4 cm, while the vestibular extension measured about 2 × 3 cm. The surface was ulcerated anteriorly in the vestibular region. On palpation, the lesion was firm, non-tender, and slightly mobile (Figure 1). Based on the clinical findings, a provisional diagnosis of pyogenic granuloma was considered, with differential diagnoses, including mucoepidermoid carcinoma, carcinoma of the right alveolus or hard palate, and pleomorphic adenoma.

**Figure 1:**
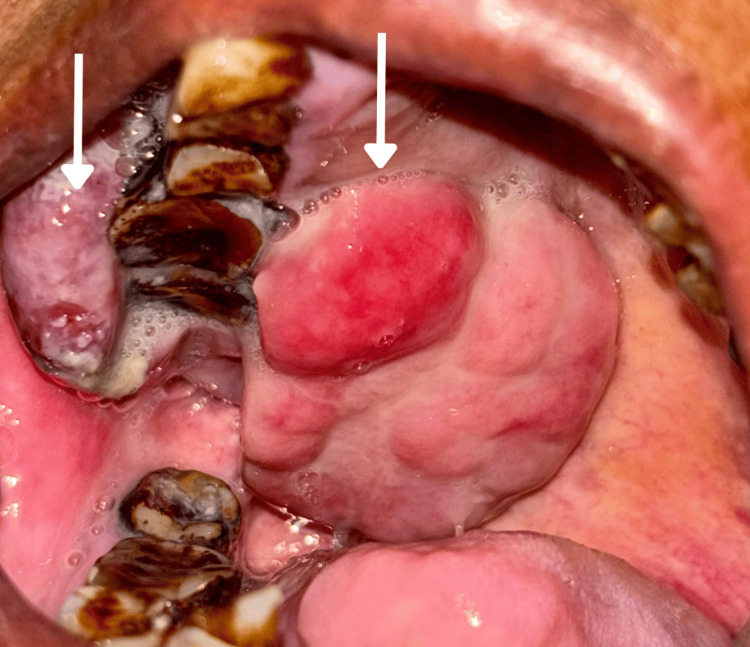
Clinical intraoral photograph Clinical intraoral photograph showing a lobulated, erythematous swelling involving the right hard palate with extension into the right maxillary gingivobuccal sulcus. Focal ulceration is visible in the vestibular region.

Radiographic investigations

A maxillary occlusal radiograph demonstrated periapical radiolucency in the region of tooth 18 and alteration of the trabecular pattern in the palatal region, with suspected areas of cortical discontinuity. Orthopantomography showed possible alveolar bone erosion adjacent to the right posterior maxillary alveolus (Figure 2). Contrast-enhanced CT of the neck revealed an ill-defined, heterogeneously enhancing lesion measuring approximately 2 × 1.2 × 1.5 cm in the right upper alveolus in relation to the second and third molars, with erosion of the alveolar ridge. The radiologic impression favored a neoplastic etiology. Contrast-enhanced CT was preferred over cone beam CT, as it was the standard imaging modality at the referring tertiary cancer center, providing additional information on soft tissue extent, lesion enhancement pattern, and regional nodal status.

**Figure 2:**
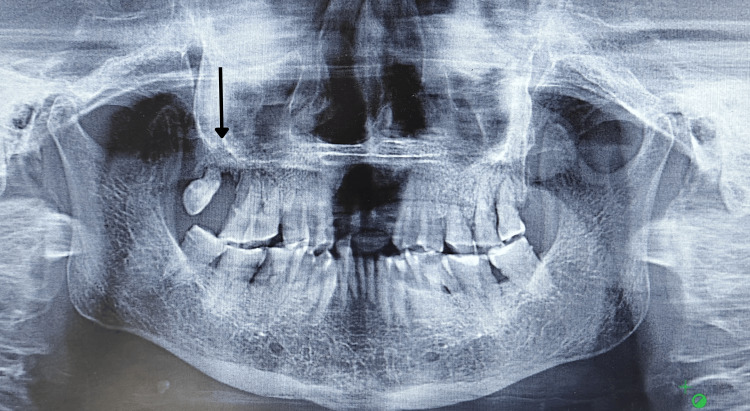
Orthopantomogram demonstrating alveolar bone erosion Orthopantomogram demonstrating irregularity and suspected erosion of the right posterior maxillary alveolar ridge corresponding to the clinical lesion, with associated missing and compromised teeth (arrow indicates area of alveolar bone erosion).

Histopathologic evaluation

Initial incisional biopsies from the right hard palate and gingivobuccal sulcus showed hyperplastic stratified squamous epithelium with surface ulceration, underlying granulation tissue, proliferating thin-walled blood vessels, oval-to-spindle cells, and a mixed inflammatory infiltrate. Immunohistochemistry for cytokeratin, p63, p40, and epithelial membrane antigen was negative; the lesion was interpreted as granulation tissue with fibroblastic proliferation, without evidence of malignancy. The non-diagnostic nature of the initial biopsies is attributed to superficial sampling, which captured only the surface granulation tissue without reaching the deeper myofibroblastic component.

Subsequent slide review and deeper sections at our institution revealed fibrous tissue with a fibrinopurulent surface component. The underlying stroma showed a prominent mixed inflammatory infiltrate of neutrophils, lymphocytes, mast cells, and plasma cells, consistent with the myxoid, vascular, and inflammatory pattern of IMT [3]. Areas of myxoid stroma containing elongated spindle-to-stellate cells with indistinct cell borders and pale eosinophilic cytoplasm were noted (Figures 3A, 3B). A focal perivascular tram-track arrangement of spindle cells was observed, reflecting the characteristic smooth muscle actin (SMA) cytoskeletal staining pattern of myofibroblasts. Scattered degenerative pleomorphic nuclei and rare mitotic figures were present, along with numerous delicate thin-walled branching blood vessels with focal red cell extravasation. 

**Figure 3:**
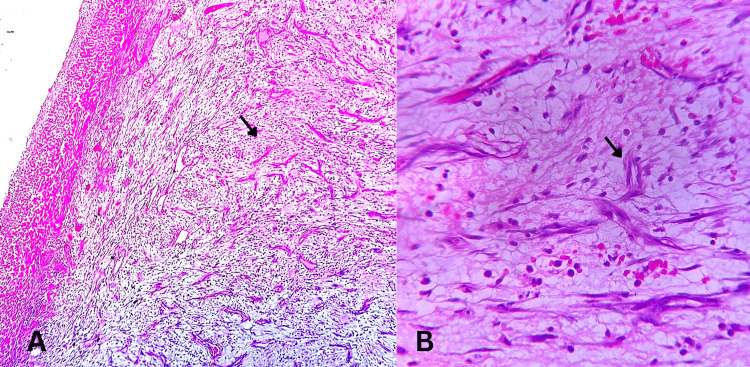
Histopathologic features of inflammatory myofibroblastic tumor (A) Low-power view (×100): Ulcerated surface with overlying fibrinopurulent exudate, underlying granulation tissue composed of proliferating thin-walled capillaries, fibroblasts, and mixed inflammatory infiltrate, with transition into myxoid stroma, consistent with the myxoid, vascular, and inflammatory pattern of inflammatory myofibroblastic tumor. (B) High-power view (×400): Myxoid stroma containing elongated spindle-to-stellate myofibroblasts with bland nuclear morphology, pale eosinophilic cytoplasm, and indistinct cell borders; prominent lymphoplasmacytic and mixed inflammatory infiltrate with thin-walled blood vessels (arrow) and focal red cell extravasation. Hematoxylin and eosin stain; original magnifications ×100 (A) and ×400 (B)

An extended immunohistochemical panel demonstrated diffuse positivity of the spindle and stellate cells for smooth muscle actin in the characteristic myofibroblastic tram-track pattern, desmin, and vimentin (Figure 4). Focal, patchy cytokeratin positivity was noted, while staining for *ALK* and CD34 was negative. The overall morphologic and immunohistochemical profile confirmed a diagnosis of *ALK*-negative IMT, myxoid pattern. 

**Figure 4:**
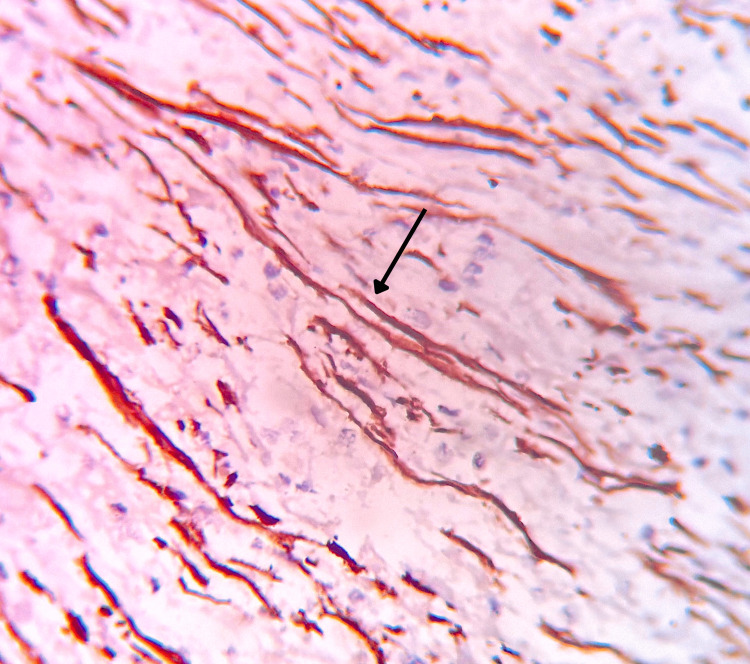
Smooth muscle actin immunohistochemistry showing tram-track staining pattern Immunohistochemical staining demonstrating diffuse positivity of tumor cells for smooth muscle actin (SMA) in the characteristic myofibroblastic tram-track pattern, visible as parallel linear brown staining along the cell membranes and cytoplasmic stress fibers of spindle cells, supporting myofibroblastic differentiation. Arrow indicates tram-track SMA-positive spindle cells (immunohistochemistry, ×400).

Treatment and follow-up

Complete surgical excision of the lesion was performed. Histopathologic examination confirmed the diagnosis of IMT with clear surgical margins. The postoperative course was uneventful. At five-month follow-up, the patient remained asymptomatic with satisfactory healing and no clinical or radiologic evidence of recurrence (Figure 5).

**Figure 5:**
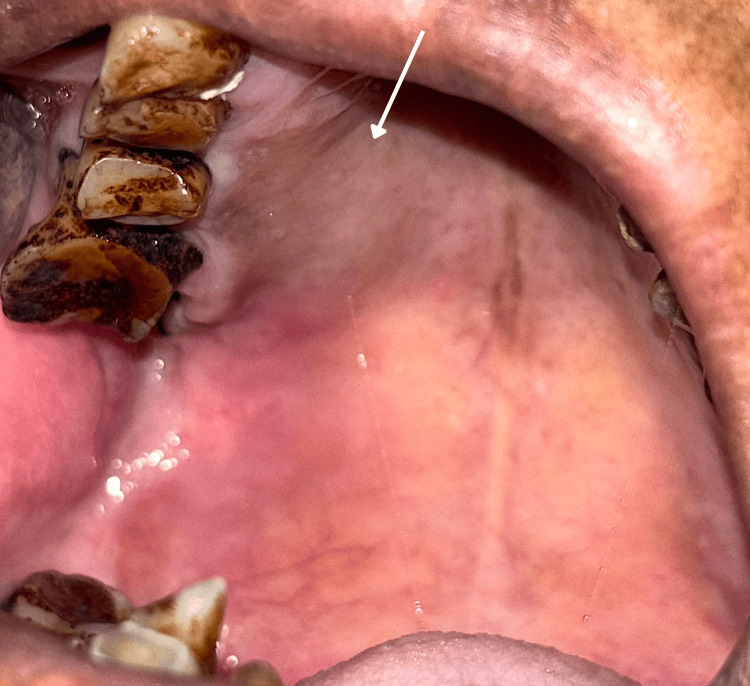
Postoperative intraoral photograph at five-month follow-up Postoperative intraoral photograph at five-month follow-up showing complete healing of the surgical site in the right hard palate region. The mucosa appears smooth and intact with no evidence of residual lesion, ulceration, or recurrence. Arrow indicates the previously affected surgical site.

## Discussion

IMT is an uncommon mesenchymal neoplasm characterized by myofibroblastic spindle cells accompanied by a variable inflammatory infiltrate. Initially regarded as a reactive process described under the umbrella of inflammatory pseudotumor, IMT is now recognized by the World Health Organization as a true neoplasm of intermediate biological potential, specifically classified as an intermediate fibroblastic and myofibroblastic tumor due to its locally aggressive behavior and rare capacity for recurrence or metastasis [10]. Although IMT most commonly involves the lung and abdominopelvic organs, its occurrence in the head and neck region is infrequent, with the oral cavity representing an exceptionally uncommon site of involvement [11]. The etiopathogenesis of IMT remains incompletely understood. While earlier theories proposed a reactive or post-inflammatory origin, the identification of recurrent *ALK* gene rearrangements and other molecular alterations has firmly established IMT as a true neoplasm. Trauma, chronic inflammation, and viral triggers have been proposed as possible initiating factors in a subset of cases [3,7].

Oral IMTs pose a significant diagnostic challenge because their clinical and radiologic features frequently overlap with those of malignant neoplasms. Rapid enlargement, ulceration, bone erosion, and ill-defined radiologic margins in the oral cavity may strongly suggest squamous cell carcinoma or soft tissue sarcoma, particularly in elderly patients with long-standing oral risk habits [3,11]. In the present case, the patient’s age, chronic betel quid chewing habit, rapid growth, ulceration, and radiologic evidence of alveolar ridge erosion resulted in a strong initial suspicion of malignancy and referral to a tertiary cancer center. The risk-habit background was a particularly confounding factor, as it is more typically associated with squamous cell carcinoma than a myofibroblastic neoplasm.

Based on the histopathologic findings, the present case conforms to Pattern 1 (myxoid, vascular, and inflammatory pattern) of the Coffin et al. classification, characterized by loosely arranged spindle-to-stellate myofibroblasts within a myxoid stroma, abundant thin-walled vascularity, and a prominent lymphoplasmacytic and mixed inflammatory infiltrate resembling granulation tissue or a reactive process [3]. This is the pattern most commonly misinterpreted as granulation tissue or reactive fibroblastic proliferation on initial small biopsies, as observed in the present case. The morphologic heterogeneity of IMT, with patterns ranging from this myxoid type to compact spindle-cell and hypocellular fibrous types, explains the initial non-diagnostic biopsies. Deeper sections and repeat sampling were essential to demonstrate the organized myofibroblastic architecture.

Immunohistochemistry plays a pivotal role in establishing the diagnosis. In the present case, tumor cells showed diffuse smooth muscle actin positivity in the characteristic myofibroblastic tram-track staining pattern, reflecting the arrangement of actin filaments along the cell membrane and cytoplasmic stress fibers of myofibroblasts, a pattern that is distinct from the block cytoplasmic staining observed in smooth muscle tumors and serves as a useful discriminator [7]. This tram-track SMA pattern is a well-recognized feature of myofibroblastic differentiation. Co-expression of desmin and vimentin further supported the myofibroblastic immunophenotype. Focal cytokeratin positivity, although potentially misleading, is a recognized finding in approximately 30% of IMTs and should not be interpreted as evidence of epithelial malignancy in the absence of diffuse epithelial marker expression or p63/p40 positivity [1,12]. Awareness of this pitfall is essential to avoid misclassification as spindle cell carcinoma.

*ALK* gene rearrangements are detected in approximately 50%-60% of all IMTs, predominantly in children and young adults, and support a neoplastic pathogenesis [7]. *ALK*-negative IMTs are more frequently encountered in adult and elderly patients. Notably, Coffin et al. reported that all metastatic IMTs in their series were *ALK*-negative, and Bakhshwin et al. found that despite earlier reports, *ALK* expression may still be detected in up to 91% of IMTs in adults ≥40 years [3,13]. The absence of *ALK* expression in the present case is therefore consistent with the patient’s advanced age, though it represents an even more challenging diagnostic scenario given the lack of this supportive immunohistochemical marker.

The differential diagnosis of oral IMT is broad and includes reactive and neoplastic spindle cell lesions [1]. Nodular fasciitis and exuberant granulation tissue may show rapid growth, myxoid stroma, and spindle cell proliferation [14]. However, these lesions are typically self-limiting, lack a persistent mass-forming architecture, and do not demonstrate the organized myofibroblastic proliferation with a sustained inflammatory background observed in IMT. Fibromatosis and fibromatosis-like lesions were also considered; however, these entities are usually paucicellular, collagen-rich, and lack a prominent inflammatory infiltrate [15]. 

Low-grade myofibroblastic sarcoma (LGMS) represented a particularly significant differential diagnosis in the present case [16]. LGMS typically demonstrates infiltrative growth, increased cellularity, cytologic atypia, and a higher mitotic index, often with minimal inflammatory infiltrate, distinguishing it from IMT [16]. Importantly, while LGMS may also show SMA positivity in a tram-track pattern, the combination of predominantly bland spindle-to-stellate cells, a conspicuous and diffuse mixed inflammatory background, rare mitotic figures, and absence of overt cytologic atypia in the present lesion favored IMT over LGMS.

Vascular tumors such as angiofibroma and hemangiopericytoma-like lesions were excluded based on the absence of a characteristic staghorn vascular pattern and CD34 negativity. Solitary fibrous tumor was ruled out by the absence of a patternless architecture and CD34 negativity [17]. Spindle cell (sarcomatoid) carcinoma was a critical exclusion in view of the patient’s age, habit history, ulceration, and radiologic bone erosion; however, the absence of diffuse epithelial marker expression, together with the presence of a prominent inflammatory milieu and myofibroblastic immunophenotype with tram-track SMA positivity, argued strongly against an epithelial malignancy [18].

Complete surgical excision remains the treatment of choice for oral IMT. Extrapulmonary IMT carries an overall recurrence rate of approximately 25%; however, oral and head-neck IMTs treated with complete excision demonstrate considerably lower recurrence rates [1,9]. The absence of recurrence at five-month follow-up in the present case is consistent with these observations and supports the importance of achieving clear surgical margins. Accurate diagnosis remains paramount in preventing unnecessary aggressive oncologic treatment.

## Conclusions

IMT of the oral cavity is an uncommon entity that can closely mimic malignant neoplasms on clinical, radiologic, and histopathologic evaluation. The present case, an *ALK*-negative IMT of the myxoid pattern involving dual oral sites in an elderly patient with risk habits, illustrates a convergence of diagnostic pitfalls: rapid enlargement and bone erosion clinically indistinguishable from malignancy, two successive non-diagnostic biopsies reflecting the morphologic heterogeneity of the myxoid histologic pattern, and focal cytokeratin positivity on immunohistochemistry that risked misclassification as spindle cell carcinoma. 

Recognition of IMT histologic subtypes, particularly the myxoid pattern, and familiarity with the characteristic myofibroblastic tram-track SMA staining pattern are essential for pathologists evaluating oral spindle cell lesions. Thorough clinicopathologic correlation, adequate tissue sampling with deeper sections, and a systematic immunohistochemical approach are necessary to establish the correct diagnosis and guide appropriate conservative surgical management. A limitation of this report is the relatively short follow-up duration of five months; long-term surveillance is recommended given the potential for recurrence, particularly in *ALK*-negative cases.
